# Effects of sedative-hypnotics on sleep quality among patients with insomnia: evidence from an observational, pre-post study in India

**DOI:** 10.1186/s12955-020-01379-z

**Published:** 2020-07-06

**Authors:** Gautam Satheesh, Sandra Puthean, Abhishek Sharma, Shiva Raj Mishra, Jeswin Jose, Sushil Kakkan, M. K. Unnikrishnan

**Affiliations:** 1 Department of Pharmacy Practice, National College of Pharmacy, Kozhikode, Kerala India; 2 Department of Global Health, Boston University School of Public Health, Boston, MA USA; 3 PRECISIONheor, Precision Value & Health, Boston, MA USA; 4 Nepal Development Society, Bharatpur-10, Chitwan Nepal; 5 University of Queensland, Brisbane, Queensland Australia; 6 Department of Psychiatry, KMCT Medical College Hospital, Kozhikode, Kerala India

**Keywords:** Insomnia, Sleep quality, Sedative-hypnotics, Zolpidem, Benzodiazepines, Quality of life, Health outcomes

## Abstract

**Background:**

Insomnia continues to be neglected globally, despite its high prevalence. Guidelines by the health regulatory agencies call for studies to evaluate the effect of sedative-hypnotics on sleep quality.

**Methods:**

We conducted a pre-post observational study to evaluate sleep quality among 186 inpatients receiving short-term oral sedative-hypnotic therapy in a tertiary care teaching hospital in Kozhikode (Kerala), India. Using Pittsburgh Sleep Quality Index_Past-Week (PSQI_PW) questionnaire, patients were interviewed upon hospital admission and at follow up after ≥1-week of sedative-hypnotic therapy. Additionally, we interviewed 36 physicians to understand the current clinical perception about sedative-hypnotics.

**Results:**

Mean (SD) age of the study patients was 59 (7.5) years. Majority (63.4%) of the patients were men. Of the various primary diagnoses for hospitalization, cardiovascular disease was the most common (22.6%, *n* = 49). Sedative-hypnotic therapy improved the mean (SD) PSQI_PW overall score by 6.79 points (pre: 12.70 (3.5) vs. post: 5.91 (2.8); *p* < 0.0001). Statistically significant improvements in sleep duration, latency, efficacy, and day dysfunction were observed. Higher proportion of study patients were prescribed benzodiazepines (73.7%) compared to zolpidem (26.3%). Patients treated with zolpidem reported higher improvements in mean overall PSQI_PW scores compared to those treated with benzodiazepines, however these differences were not statistically significant upon adjusting for age, gender and primary diagnosis for hospitalization. Qualitative interviews indicate that that physicians consider zolpidem to be safer and more efficacious.

**Conclusions:**

In our study, sedative-hypnotic therapy helped improve sleep quality among the hospitalized patients. More studies evaluating the comparative efficacy and safety of zolpidem vs. benzodiazepines – including among patient groups with varying demographic and clinical characteristics – are needed. India must develop evidence-based treatment guidelines to inform the clinical practice around the use of sedative-hypnotics.

## Background

Insomnia continues to be overlooked despite its association with life threatening comorbidities like cardiovascular diseases and diabetes. Addressing the deleterious effects of insomnia poses a challenging task, especially in India, considering patients’ attitude about the disease and the suboptimal healthcare system [1]. A recent report claimed that over 90% of the Indians fail to receive adequate sleep, with up to 58% believing that their work efficiency is affected by poor sleep quality and 11% falling asleep during work. However, only 2% of those with sleep-deprivation discuss their insomnia with a physician [2]. Mental disorders are a strong social stigma in India; insomnia often goes underreported like other mental disorders (such as anxiety and depression) because consulting a psychiatrist is often considered a taboo [3, 4]. A global survey entitled “*Better Sleep, Better Health: A global look at why we’re still falling short on sleep*” – conducted across 13 countries including the USA and UK – reported a very high prevalence of poor sleep in India [1]. In South India, the urban population seems to be more predisposed to difficulty in initiating and maintaining sleep than their rural counterparts [5]. Lack of awareness, coupled with inadequate health seeking behaviour, possibly underemphasizes the importance of healthy sleep [5]. The prevalence of insomnia is also high in North India – with over 75% patients reporting poor sleep quality upon hospital admission [6].

Sleep-wake cycle is greatly disrupted with ageing. Sleep apnoea and insomnia appear to be the major sleep problems among the elderly; approximately 50% of the elderly report difficulty initiating as well as maintaining sleep [7]. Alteration of sleep architecture includes longer peripheral stages of sleep (N1 and N2) and shorter stages of deep sleep (N3) or REM sleep. Insomnia in geriatric population is frequent, yet unresolved [8]. Although many diseases often contribute to insomnia, one cannot generalize that all comorbidities have a deleterious effect on sleep [9]. On the contrary, inadequate sleep is associated with higher risk as well as poorer prognosis of chronic diseases such as diabetes and cardiovascular diseases [10, 11]. Moreover, poor subjective sleep quality and inadequate sleep duration adversely affect the overall health-related quality of life [12]. In particular, sleep quality and duration are often compromised among the hospitalized patients due to pain, acute illness, anxiety, medical intervention, environmental noise and lights [13, 14].

Sedative-hypnotic drugs (primarily benzodiazepines (BZDs) and non-BZDs) are the mainstay for insomnia. To ensure the rational use of sedative-hypnotics, regulatory bodies (such as UK’s National Institute for Health and Care Excellence) have recommended further studies to evaluate the impact of hypnotics on the improvement of sleep quality and daytime sedation. Pittsburgh Sleep Quality Index (PSQI), a standardized questionnaire by the University of Pittsburgh, is a reliable tool to evaluate sleep quality [15]. Employing this questionnaire, we evaluated the sleep quality of inpatients undergoing short-term oral sedative-hypnotic therapy in a tertiary care hospital setting in South India.

## Methods

This observational, pre-post study was designed to assess the sleep quality among in-patients undergoing oral sedative-hypnotic therapy. We obtained a modified one-week version of Pittsburgh Sleep Quality Index Past Week (PSQI_PW) from the University of Pittsburgh. We conducted in-depth patient interviews on the day of hospital admission (pre-score) and on the day of discharge or 7 days after hospital admission (post-score), whichever happened earlier. We also report on the factors influencing sleep quality, the sedative-hypnotic prescribing pattern, and patient-reported adverse effects of sedative-hypnotic drugs. We use the terms 'therapy' and 'treatment' interchangeably in this manuscript.

### Study setting and patient sample

The study was conducted in an 800 bedded tertiary care teaching hospital in Kozhikode (Kerala state), India during November 2017 to May 2018. We enrolled all the inpatients (*n* = 186) who complained of difficulty initiating and/or maintaining sleep (insomnia) and were prescribed oral sedative-hypnotic on the day of hospital admission. These patients had varying primary diagnoses from various specialty departments. Patients with chronic insomnia (sleep trouble occurring at least 3 nights per week for at least 3 months) or those on long-term sedative-hypnotics therapy were excluded [16]. We also excluded all children, pregnant women, and the patients undergoing surgery.

### Data collection and statistical analysis

We collected patient scores – based on PSQI_PW instructions – to assess the four variables of sleep quality i.e. sleep duration, sleep efficiency, latency and day dysfunction. See Table 1. Individual scores of these variables were added up to calculate the total pre- and post-score (i.e. before and after the sedative-hypnotic therapy, respectively), ranging from 0 (better) to 21 (worse) for each patient. We also interviewed the patients (or caretaker for psychiatric patients) using PSQI_PW questionnaire. The patient case files were followed up each day to accurately record the occurrence of adverse events.

**Table 1:** Sleep variables in Pittsburgh Sleep Quality Index Past Week (PSQI_PW) questionnaire

Sleep variables	Definition	Scale
PSQI_PW	Global score (overall sleep quality)	0 (better) to 21 (worse)
Sleep Duration	Hours of sleep every night	0 (better; if duration ≥7 h) to 3 (worse; if duration < 5 h)
Sleep Latency	Minutes to accomplish full transition from wakefulness to sleep	0 (better; if latency ≤15 min) to 3 (worse; if latency > 60 min)
Day Dysfunction due to sleepiness	Inability to stay awake and keep up enthusiasm during daytime.	Binary: yes / no
Sleep Efficiency	The percentage of time in bed that one is asleep	Range: 0 (better) to 3 (worse)

Following a week long oral sedative-hypnotic therapy, we re-interviewed the patients to calculate the post-scores. We conducted follow up interviews via phone for the patients discharged within 7 days of hospital admission. Differences in mean sleep duration, mean sleep latency and mean sleep efficiency were compared. Due to high correlation between these continuous measures at two measurement times, a linear mixed model was used at alpha significance level of 0.05. An unstructured correlation structure was chosen because of limited measurement times (pre-post), and we used the Ken-Ward-Roger approximation method to compute denominator degrees of freedom [17]. Additionally, patient-reported adverse effects related to sedative-hypnotic therapy were recorded. McNemar’s statistical test was performed to compare the pre and post categorical values for day dysfunction. We employed SAS 9.4 for all the analyses.

We also conducted in-depth interviews with 36 physicians identified through convenience sampling, in order to understand the current clinical perception of sedative-hypnotic use.

## Results

### Pre-treatment sleep quality

Among the 186 patients enrolled in the study, the mean (SD) age was 59 (7.5) years (range: 40–70 years) and the majority were men (63.4%, *n* = 118). Patients were hospitalized primarily for cardiovascular diseases (22.6%, *n* = 49) followed by respiratory diseases (19.9%, *n* = 37). See Table 2.

**Table 2:** Patient demographics and Sleep Quality on the day of hospital admission

	Study patients (*N* = 186)	PSQI_PW = 5–10 i.e. poor sleep (*N* = 53)	PSQI_PW > 10 i.e. very poor sleep (*N* = 133)
**Gender (%, n)**			
Male	63.4% (*n* = 118)	17.7% (*n* = 33)	45.7% (*n* = 85)
Female	36.6% (*n* = 68)	10.8% (*n* = 20)	25.8% (*n* = 48)
**Age, Mean (SD)**	59.2 (7.5)	58.0 (6.2)	59.3 (7.9)
**Age Group (%, n)**			
40–50 years	23.1% (*n* = 43)	4.8% (*n* = 9)	18.3% (*n* = 34)
50–60 years	48.4% (*n* = 90)	18.3% (*n* = 34)	30.1% (*n* = 56)
60–70 years	28.5% (*n* = 53)	5.4% (*n* = 10)	23.1% (*n* = 43)
**Primary reason for hospitalization (%, n)**			
Cardiovascular disease	22.6% (*n* = 42)	10.2% (*n* = 19)	12.4% (*n* = 23)
Respiratory diseases	19.9% (n = 37)	3.8% (*n* = 7)	16.1% (*n* = 30)
Infectious diseases	13.4% (*n* = 25)	2.7% (*n* = 5)	10.7% (*n* = 20)
Psychiatric disorders	15.1% (*n* = 28)	2.7% (*n* = 5)	12.4% (*n* = 23)
Fracture	7.5% (*n* = 14)	1.1% (*n* = 2)	6.4% (*n* = 12)
Urologic diseases	14.0% (*n* = 26)	5.4% (*n* = 10)	8.6% (*n* = 16)
Other diseases	7.5% (*n* = 14)	2.7% (*n* = 5)	4.8% (*n* = 9)

**Mean (SD) [range] overall PSQI_PW pre-treatment score: 12.70 (3.5) [range: 5.0 to 19.0].**

When subjectively evaluated before treatment, pre-treatment PSQI_PW scores were greater than 10 for 71.5% (*n* = 133), indicating very poor sleep quality. This was most common among patients with respiratory diseases (16.1%), followed by those with cardiovascular (12.4%) and psychiatric disorders (12.4%). The mean pre-treatment PSQI_PW score was 12.7 (SD: 3.5) [range: 5–19]. See Table 2.

On the day of hospital admission, 58% (*n* = 108) of the patients experienced difficulty initiating sleep (sleep latency > 15 min), 68% (*n* = 127) reported sleeping < 5 h, and 39.3% (*n* = 73) experienced some level of day dysfunction due to sleepiness. In the pre-treatment interviews, hospitalized patients reported a mean sleep duration of 4.70 h and a mean sleep latency of 22.50 min (Table 3). The mean (SD) sleep efficiency score was 2.28 (0.56). See Table 3.

**Table 3:** Sleep quality among study patients: pre and post sedative-hypnotic treatment

Variable	Pre-treatment	Post-treatment	Pre-post comparison	
			Mean difference (95%) CI	*P*-value (Bonferroni-adjusted)
Mean (SD) sleep duration, hours*	4.70 (1.18)	6.90 (1.03)	2.2 (2.0, 2.4)	< 0.001
Mean (SD) sleep latency, minutes*	22.50 (8.50)	7.90 (4.30)	14.6 (12.5, 16.7)	< 0.001
Mean (SD) Sleep efficiency score*	2.28 (0.60)	0.63 (0.63)	−1.7 (− 1.5, − 1.8)	< 0.001
**Experience Day Dysfunction due to sleepiness** (Percentage, yes) **┼**	39.3% (*n* = 73)	24.2% (*n* = 45)	─	0.0001

*The mean score differences were compared using a linear mixed model, adjusting for age, gender and primary diagnosis for hospitalization. **┼** McNemar’s test was performed for pre-treatment and post-treatment comparison patients experiencing day dysfunction

Prior to sedative-hypnotic treatment, about 80% (*n* = 149) reported abrupt awakening at night - mostly due to pain (14%) and dyspnoea (10.8%). About 10% patients experienced severe broken sleep without an identifiable cause and reported waking up multiple times at night. Majority of the sleep disturbances could be attributed to an underlying health condition i.e. pain among fracture patients, anxiety among cardiovascular disease patients, and dyspnea in patients with respiratory diseases. See Additional File 1: Figure S1.

### Sedative-hypnotic medication use among study patients

Most study patients (73.7%; *n* = 137) received BZDs i.e. alprazolam, clonazepam, lorazepam and nitrazepam. Alprazolam (40.3%, *n* = 75) was the most frequently prescribed BZD – especially to patients with cardiovascular diseases and urologic diseases. Clonazepam (19.35%, *n* = 36) was the preferred alternative to alprazolam. Lorazepam (11.9%, *n* = 22) was the preferred hypnotic for psychiatric patients. Nitrazepam was least frequently prescribed (2.2%).

**Fig. 1:**
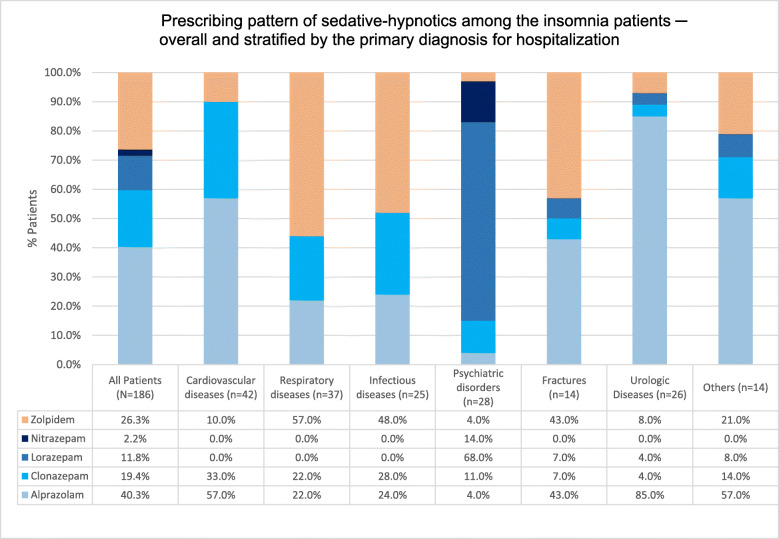
Prescribing pattern of sedative-hypnotics among the insomnia patients by primary diagnosis groups

In contrast, only 26.3% (*n* = 49) received non-BZD hypnotics i.e. zolpidem. Zolpidem was preferred for patients with respiratory diseases, probably due to risk of respiratory depression associated with BZDs. Other hypnotics i.e. Z-drugs (zaleplon, zopiclone) and melatonin receptor agonist (ramelteon) were not prescribed to any study patient. See Fig. 1.

### Post-treatment sleep quality

Adjusting for age and gender, there were significant improvements in mean sleep duration that increased from 4.7 h to 6.9 h [mean difference (95% CI): 2.2 (2.0, 2.4) hours; *p*-value < 0.001]. The mean sleep latency decreased from 22.5 min to 7.9 min [mean difference (95% CI): 14.6 (12.5, 16.7), *p*-value < 0.001]. We observed an improvement in sleep efficiency [scale: 0 (better) to 3 (worse)]; mean (SD) scores decreased from 2.28 (0.56) to 0.63 (0.63) i.e. a mean difference (95% CI) of − 1.7 (− 1.5, − 1.8). The proportion of patients experiencing day dysfunction also reduced from 39.3% at the time of admission to 24.2% (*p*-value = 0.0001). See Table 3 and Additional File 1: Table S1 and S2 for details.

Since six statistical tests were used in the pre and post treatment comparison (i.e. one each for sleep duration, latency, efficiency, day dysfunction, primary diagnosis and medication), we applied Bonferroni correction by multiplying *p*-value by 6.

Upon sedative-hypnotic therapy, the overall mean (SD) PSQI total score improved by 6.79 points (pre: 12.70 (3.5) vs. post: 5.91 (2.8); *p* < 0.0001) after 7 days. Comparing patient groups by primary diagnosis for hospitalization, the mean PSQI_PW score improvement was the highest among patients with fractures (pre: 15.71 vs. post: 5.79; *p* < 0.0001) followed by those with infections (pre: 13.28 vs. post: 4.96; p < 0.0001). Difference in the pre-post PSQI_PW scores was relatively lower but were statistically significant in patients with psychiatric disorders (pre: 13.75 vs. post: 7.25; p < 0.0001) and respiratory diseases (pre: 13.83 vs. post: 7.46; p < 0.0001). Patients with cardiovascular diseases showed least improvement in sleep quality (pre: 11.01 vs. post: 5.25; p < 0.0001). See Table 4(a).

**Table 4:** Unadjusted mean PSQI_PW scores pre and post treatment – stratified by primary diagnosis for hospitalization and sedative-hypnotic drug type

	Mean Pre-Score (SD)	Mean Post-Score (SD)	Pre-Post change (%)	Mean difference (95% CI)	p-value (Bonferroni-adjusted)
**4(a). Primary diagnosis**					
Cardiovascular diseases	11.01 (3.27)	5.25 (2.11)	−5.76 (52.3%)	5.6 (4.4,6.7)	< 0.001
Respiratory diseases	13.83 (3.86)	7.46 (3.01)	−6.37 (46.1%)	6.7 (5.3,8.1)	< 0.001
Infectious diseases	13.28 (3.27)	4.96 (2.81)	−8.32 (62.7%)	8.3 (6.6,10.1)	< 0.001
Psychiatric disorders	13.75 (3.01)	7.25 (2.85)	−6.50 (47.3%)	6.5 (5.1,7.9)	< 0.001
Fractures	15.71 (3.05)	5.79 (2.55)	−9.93 (63.2%)	9.9 (7.8,12.0)	< 0.001
Urologic diseases	11.31 (2.41)	5.38 (2.68)	−5.92 (52.3%)	5.9 (4.6,7.3)	< 0.001
Other diseases	11.21 (3.81)	3.93 (2.13)	−7.29 (65.0%)	7.3 (4.8,9.7)	< 0.001
**4(b). Sedative-hypnotics**					
Alprazolam	11.76 (3.51)	5.31 (2.70)	−6.45 (54.8%)	6.5(5.6,7.3)	< 0.001
Clonazepam	12.00 (3.82)	5.83 (2.68)	−6.17 (51.4%)	6.2 (5.0,7.3)	< 0.001
Zolpidem	14.32 (3.02)	6.41 (2.87)	−7.91 (55.2%)	7.9 (6.7,9.1)	< 0.001
Lorazepam	13.32 (3.24)	6.73 (2.99)	−6.59 (49.5%)	6.6 (4.8,8.4)	< 0.001
Nitrazepam	13.25 (1.26)	7.00 (2.94)	−6.25 (47.2%)	6.3 (2.0,10.5)	0.0894

Table 4(b) reports mean pre- and post-treatment PSQI_PW scores among patients stratified by administered sedative-hypnotic drug. Among patients who received BZDs, the mean differences in PSQI_PW score ranged from − 6.17 (clonazepam) to − 6.59 (lorazepam). The pre-post differences (i.e. improvements) were statistically significant for all BZD patient sub-groups, except those who received nitrazepam. Patients who received zolpidem experienced statistically significant improvements in sleep quality; the pre-post mean difference in PSQI_PW score was − 7.91 (pre: 14.32 vs. post 6.41; *p* < 0.001).

Overall, the mean pre and post PSQI_PW scores were 12.7 and 5.9 (percent improvement: 53%), respectively. For comparisons among patient sub-groups, the mean pre and post treatment PSQI_PW scores were compared using a linear mixed model, adjusting for age and gender.

**Fig. 2:**
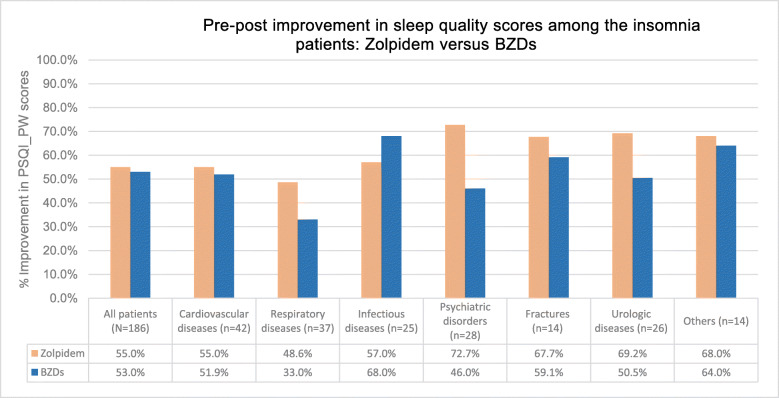
Pre-post improvement in sleep quality scores among the insomnia patients: Zolpidem versus BZDs

Table 5 compares the mean PSQI_PW improvements in patients administered BZDs versus zolpidem. In the unadjusted analysis, the percentage pre-post improvement among the study patients – overall and in each primary diagnosis group except infectious diseases – was relatively higher among those treated with zolpidem (Fig. 2). However, upon adjusting for age, gender and primary diagnosis for hospitalization, the difference in mean PSQI_PW improvements among zolpidem and BZDs groups were not statistically significant.

**Table 5:** PSQI_PW improvements in patients administered zolpidem and BZDs – adjusting for age, gender and primary diagnosis for hospitalization

Primary Diagnosis	Zolpidem (*N* = 49 patients)				BZDs (*N* = 137 patients)				Zolpidem vs. BZDs	
	*Pre-treatment*	*Post-treatment*	*Unadjusted Mean Difference (95% CI)*	*p-value*	*Pre-treatment*	*Post-treatment*	*Unadjusted Mean Difference (95% CI)*	*p-value*	*Difference in mean difference*^a^*(95% CI)*	*p-value*
Cardiovascular diseases	16.5	9.0	-7.5 (-10.3, -4.7)	<0.001	10.4	4.9	-5.5 (-6.3, -4.9)	<0.001	*-1.5 (-3.9,0.9)*	0.20
Respiratory diseases	14.4	7.4	-7.0 (-8.2, -5.9)	<0.001	13.1	7.5	-5.4 (-7.3, -3.6)	<0.001	*-1.6 (-3.7,0.4)*	0.12
Infectious diseases	13.5	5.8	-7.7 (-9.3, -6.2)	<0.001	13.1	4.2	-8.8 (-10.8, -6.8)	<0.001	*1.0 (-1.4,3.4)*	0.39
Psychiatric disorders	11.0	3.0	-8.0 (-8.0, -8.0)	0.0036	13.8	7.4	-6.4 (-7.7, -5.2)	<0.001	*-2.1 (-10.2,6.0)*	0.59
Fractures	15.5	5.0	-10.5 (13.4, -7.6)	<0.001	15.8	6.4	-9.4 (-12.0, -7.0)	<0.001	*1.2 (-1.4,3.9)*	0.33
Urologic diseases	13.0	4.0	-9.0 (-21.7, 3.7)	<0.001	11.1	5.5	-5.7 (-6.8, -4.5)	<0.001	*-2.6 (-7.1,1.9)*	0.23
Others	13.3	4.3	-9.0 (-13.3, -4.7)	<0.001	10.6	3.8	-6.8 (-9.1, -4.5)	<0.001	*-2.1 (-7.0,2.9)*	0.37
All patients	14.3	6.4	-7.9 (-8.6, -7.1)	<0.001	12.1	5.7	-6.4 (-6.9, -5.9)	<0.001	*-1.5 (-2.6,-0.5)*	0.0028

^a^Differences in mean differences after adjusting for age, gender and primary diagnosis for hospitalization. See Additional File 1: Table S3 for distribution of age, gender and primary diagnosis for hospitalization among patients in the two drug groups

Overall, 10.2% (*n* = 19) patients reported adverse effects following initiation of sedative-hypnotic therapy, of which 74% were aged > = 60 years. Of the patient-reported adverse effects, daytime drowsiness was the most common (*n* = 6) followed by early-morning awakening (*n* = 3), nightmares (n = 3) and dysgeusia i.e. altered taste (*n* = 2). None of the patients encountered falls or minor accidents. Patients treated with zolpidem did not report early morning awakening or daytime sedation (Additional File 1: Table S4).

For all the study patients, the short-term sedative-hypnotic therapy dosing was tapered and discontinued. While this would reduce any risk of rebound insomnia, 7% (*n* = 13) reported persistent insomnia following discontinuation.

### Qualitative results

To understand the physicians’ standpoint with respect to the current dilemma in choosing the right sedative-hypnotic, we conducted qualitative interviews with the physicians using a semi-structured questionnaire. Of the 36 physicians surveyed from various speciality departments, 72% (*n* = 26) preferred zolpidem for its higher (perceived) efficacy than BZDs in improving sleep quality. Furthermore, 53% (*n* = 19) physicians believed that zolpidem is also safer than BZDs for inpatients.

However, prescribers’ opinion is inconsistent with the observed prescribing pattern of sedative-hypnotics in the hospital, with BZD being more frequently prescribed.

“*BZDs often cause respiratory depression in susceptible individuals (patients with respiratory diseases). When such patients complain of insomnia, Z-drugs seem to be the best treatment option.*” - Pulmonologist A

This opinion however varies from one speciality department to another. A psychiatrist seems more likely to prescribe BZDs, owing to its anxiolytic effects. Zolpidem, on the other hand - being devoid of anxiolytic effects – is less preferred in such circumstances.

“*Most of the patients who report poor sleep also complain of anxiety at bedtime, especially prior to the day of a major diagnostic procedure. Tackling anxiety with a BZD appears to be more prudent than merely inducing sleep.*” - Cardiologist X

Some physicians told that they also choose BZD because it is less expensive than zolpidem. Physicians, especially in the lower and middle income countries like India, often find the need to consider patients’ ability to afford prescription medicines [18].

## Discussion

We found that inpatients who complained of trouble initiating or maintaining sleep had poor or very poor sleep quality scores. Our findings are in accordance with previous reports of the untreated burden of insomnia among inpatients, especially the elderly [8, 9, 19]. A previous study in Brazil reported that abrupt awakenings among inpatients were primarily caused by comorbid conditions, uncomfortable bedding, nursing routine, fear, pain, breathlessness and room temperature. Patients also attributed insomnia to the comorbid condition such as fracture-induced pain, lung disease induced cough, or dyspnoea [19]. Poor sleep not only affects overall health, but also has negative impact on one’s appearance, mood, motivation and concentration [1].

In our study, short-term oral sedative-hypnotic therapy improved sleep quality among majority of the inpatients who experienced insomnia. Comorbidity-induced insomnia benefited from sedative-hypnotic therapy; maximally in patients with hospitalizations due to fractures. The small proportion (7%) of the study population who reported persistent insomnia after sedative-hypnotic discontinuation also reported very poor prognoses; neither did their existing health condition or underlying diseases improve upon hospitalization, nor did they benefit much from sedative-hypnotic therapy.

Literature indicates that sleep quality is a proxy prognostic marker for comorbid conditions [20–23]. Therefore, timely diagnosis and management of insomnia – with lifestyle/behavioural modifications and/or sedative-hypnotics therapy as appropriate – is important. With respect to sedative-hypnotic therapy, the choice of drug, dose frequency and duration of therapy must be customized to each patient’s characteristics and condition of severity [24]. However, in India, there is lack of clinical guidelines for prescribing of sedative-hypnotics among patients with varying diagnoses and circumstances. Our qualitative interviews indicate that utilization of short-term sedative-hypnotic therapy in patients with comorbidity-induced insomnia is largely driven by physician’s judgement.

The treatment of insomnia remains somewhat unchanged for decades, ever since the introduction of BZDs in the 1960s and Z-drugs in the 1990s. From 2006 through 2011, zolpidem prescriptions underwent a steady rise in the US [25]. Europe has witnessed a decline in BZD prescriptions with a corresponding rise in Z-drug prescriptions. This trend continues in spite of contrary guidelines issued by the UK’s National Institute for Health and Care Excellence (NICE), which favour the least expensive hypnotic i.e. BZDs [26–28]. The European Guidelines for Diagnosis and Treatment of Insomnia (2017) recommends BZD and Z-drugs as first line pharmacotherapy choice, but does not mention the differential impact of these two drugs on sleep quality [29].

Although zolpidem went on to become the most prescribed sedative-hypnotic in the US and UK over the last decade, little has this transition affected the practice in India [30, 31]. Our findings are concordant with most of the recent drug utilization evaluation studies in India, in which BZDs continue to be preferred largely over Z-drugs [31, 32]. Only 26% of the patients were prescribed zolpidem, the only Z-drug prescribed.

Although some literature hints the superiority of zolpidem over BZDs in terms of physician preference, the overall comparative evidence regarding sleep quality is inconclusive [33, 34]. In our analyses comparing zolpidem and BZDs, while zolpidem led to greater improvement in overall sleep quality among the patient, the differences in improvement were not statistically significant upon adjusting for age, gender and primary diagnosis for hospitalization. Our qualitative interviews suggest that majority of the physicians support zolpidem; however, introducing a change in the existing practice is likely to be slow because junior doctors tend to follow their seniors, who have been habituated to prescribing BZDs for decades. The tendency to follow key opinion leaders is likely to be driven by the ‘prestige-based hierarchy’ – prevailing in India [35]. Thus, individual clinical judgements of key opinion leaders appear to override clinical efficacy when the practice is primarily physician-dominated.

The inclination towards BZDs persists in India possibly due to the paucity of literature supporting the superiority of Z-drugs over BZDs. As mentioned earlier, India neither possesses nor follows standardized treatment guidelines that govern the use of sedatives. The poorly understood concept of safety and adverse effect profile of Z-drugs vs. BZDs presents a problem especially among geriatric patients and other high risk groups [36]. Nevertheless, with physicians in both the US and UK preferring zolpidem over the years, the prevailing popularity of BZDs in India is unanticipated, given the fact that Indian pharmaceutical regulations are preferentially biased towards ‘imported’ rules of practice.

On another note, poor marketing practices might have also contributed to zolpidem’s low utilization in India. A controversial advertisement campaign by Abbott Pharmaceuticals in 2011 – aiming to promote its brand of zolpidem in India – evoked widespread criticism by doctors. While the pharma-giant probably overexploited the findings of a study that associated poor sleep with increased mortality due to cardiovascular disease, the doctors pointed out that the practice of ‘pill popping’ is more harmful [37].

Cost concerns do play a vital role in the choice of drugs, especially in the rural and low socioeconomic status areas in India. BZDs – being relatively cheaper – are thus preferred over zolpidem and other Z-drugs. Despite the fact that this pattern in India complies with the NICE recommendations of prescribing hypnotic of minimal cost, sleep quality is not a factor to be neglected. With India passing The Mental Healthcare Act of 2017, ailments mentioned in the *Diagnostic and Statistical Manual of Mental Disorders, 5th edition* (DSM-V) become eligible for coverage under India’s health insurance policies [38]. As our qualitative results suggest, the choice of the sedative-hypnotic must also take into account the patients’ underlying diseases. This creates the compelling need for appropriate evidence-based treatment guidelines for sleep disorders among inpatients. We recommend more studies (such as prospective trials and/or real-world studies) evaluating the comparative efficacy and safety of zolpidem vs. benzodiazepines among patient groups with varying demographic and clinical characteristics, in order to inform treatment guidelines and clinical practice.

Our study, however, has few limitations. Our analyses did not adjust for some potential confounders – such as patient’s health status, severity of underlying condition and other comorbidities – that may influence the outcomes of sedative-hypnotic drugs in management of insomnia. Furthermore, our findings from a single tertiary healthcare centre in Kerala state cannot be extrapolated to other Indian states with different health indicators, disease burden, patient demographics and socio-economic status.

## Conclusions

In conclusion, with sleep quality being an important prognostic marker during hospitalization, lack of standard guidelines for utilization as well as continuous monitoring of the efficacy of sedative-hypnotics needs to be addressed. Our study suggests the possible superiority of zolpidem over BZDs in improving sleep quality in hospitalized patients, however these results are not conclusive. We underline the need of more clinical studies comparing the impact of zolpidem and BZDs on sleep quality in order to inform clinical guidelines and promote evidence-based use of sedative-hypnotics.

## Supplementary information

**Additional file 1.** Supplementary information on patient demographics and sleep quality.

## Data Availability

Data can be availed upon reasonable request to the authors.
