# Supplementary material for: Effects of sedative-hypnotics on sleep quality among patients with insomnia: evidence from an observational, pre-post study in India
Source: Health Qual Life Outcomes. 2020 Jul 6;18:212. doi: 10.1186/s12955-020-01379-z (PMC7336471; doi:10.1186/s12955-020-01379-z)

Additional File

**Table S1: Subjective Sleep Quality pre and post sedative-hypnotic therapy**		
**Subjective Sleep Quality**	**Pre-treatment**	**Post-treatment**
Very Good	0.5% (n=1)	43.5% (n=81)
Fairly Good	40.3% (n=75)	50.5% (n=94)
Fairly Bad	43.5% (n=81)	5.9% (n=11)
Very Bad	15.6% (n=29)	--

**Table S2: Sleep variables among study patients: pre and post sedative-hypnotic therapy**			
**Variable**	**Pre-treatment**	**Post-treatment**	**P-value (Bonferroni-adjusted)**
**Sleep duration┼**			
More than 7 hours	2.7% (n=5)	64% (n=119)	<0.001
5 – 7 hours	29% (n=54)	28.5% (n=53)	
3 – less than 5 hours	51.6% (n=96)	7.5% (n=14)	
Less than 3 hours	16.7% (n=31)	--	
**Sleep latency┼**			
Less than 15 minutes	41.9% (n=78)	98.4% (n=183)	<0.001
15-30 minutes	37.1% (n=69)	1.6% (n=3)	
30-60 minutes	11.8% (n=22)	--	
60 minutes or more	9.1% (n=17)	--	

┼ The mean score difference were compared using a linear mixed model, controlling for age, gender and primary diagnosis for hospitalization. Since six statistical tests were used in the pre and post intervention comparison (i.e. one each for sleep duration, latency, efficiency, days dysfunction, primary diagnosis and medication), we applied Bonferroni correction by multiplying p-value by 6.

Table S3: Distribution of age, gender and primary diagnosis for hospitalization among study patients – stratified by the sedative-hypnotic drug use						
**Primary diagnosis**	**Benzodiazepines (N=137)**			**Zolpidem (N=49)**		
	**Total patients**	**Age Mean (SD)**	**% Females**	**Total patients**	**Age Mean (SD)**	**% Females**
Cardiovascular disease	38	60.0 (6.3)	28.9	4	61.3 (4.5)	0.0
Respiratory diseases	16	61.3 (4.0)	31.3	21	64.0 (6.9)	23.8
Infectious diseases	13	59.4 (6.4)	46.2	12	59.7 (6.8)	50.0
Psychiatric disorders	27	52.6 (6.7)	55.6	1	70.0	0.0
Fracture	8	56.8 (9.3)	37.5	6	58.3 (11.8)	83.3
Urologic diseases	24	57.1 (7.1)	20.8	2	64.0 (7.1)	50.0
Other diseases	11	63.5 (5.7)	45.5	3	62.3 (12.4)	33.3

Table S4: Patient-reported side effects following sedative-hypnotic therapy						
**Reported side effect**	**Frequency**	**Alprazolam**	**Clonazepam**	**Lorazepam**	**Nitrazepam**	**Zolpidem**
Daytime drowsiness	3.2% (n=6)	1	2	2	1	–
Dizziness	1.6% (n=3)	1	–	1	–	1
Dry Mouth	0.5% (n=1)	–	–	–	–	1
Dysgeusia	1.1% (n=2)	–	–	–	–	2
Early awakening	2.1% (n=4)	–	2	2	–	–
Nightmare	1.6% (n=3)	1	1	–	–	1

Figure S1: Reasons for nightime sleep disturbance among patients

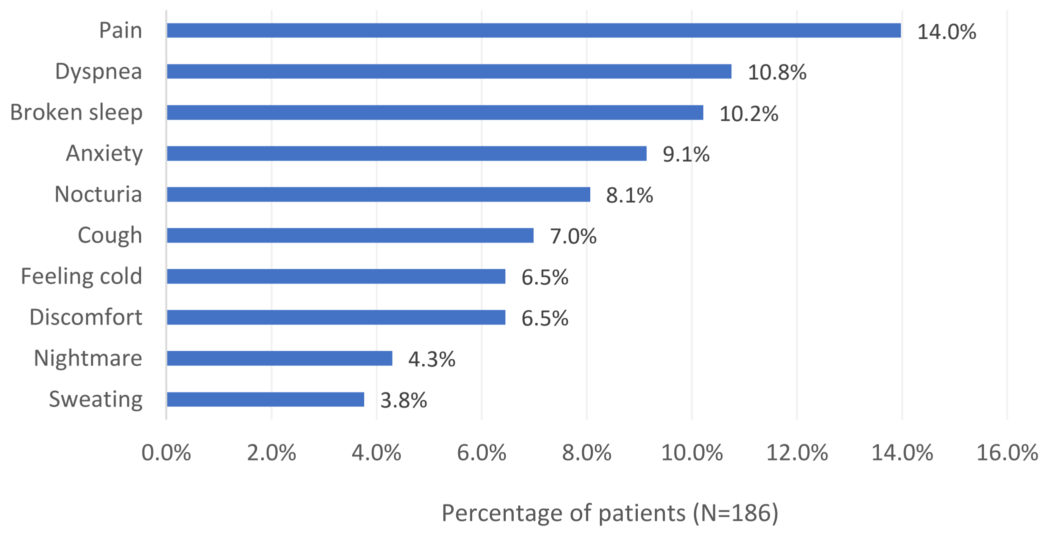

Supplement: Supplementary file 1 — Additional file 1. Supplementary information on patient demographics and sleep quality. [file 12955_2020_1379_MOESM1_ESM.pdf]
